# The impact of emotional intelligence on conflict management styles used by Saudi nurse managers—a cross sectional, correlational study

**DOI:** 10.3389/fpsyg.2025.1630530

**Published:** 2026-01-12

**Authors:** Wdad Alanazy, Sharifah Khalid S Alzamil

**Affiliations:** 1 Department of Maternity and Pediatric Nursing, College of Nursing, Majmaah University, Al Majmaah, Saudi Arabia; 2 Department of Nursing Public Health, Ministry of Health Alahssa Branch, AlHassa, Saudi Arabia

**Keywords:** emotional intelligence, conflict resolution, nurse managers, Saudi Arabia, work place

## Abstract

**Background:**

Two very important ideas for fostering harmony in the workplace are conflict management and emotional intelligence (EI). For nurse managers, controlling one’s emotions and knowing how to handle disagreement are critical skills.

**Objective:**

The purpose of this research is to evaluate how emotional intelligence (EI) affects conflict resolution techniques and how it can help Saudi Arabian nurse managers.

**Methods:**

A Correlational, quantitative cross-sectional design was employed in the study. Five regions in Saudi Arabia were selected for 500 Saudi nurse managers using convenience sampling. The study was conducted for between November 2023 and January 2024. The Vanderpol Emotional Intelligence Scale and the Rahim Organisational Conflict Inventory-II were the two items that the participants answered online. Descriptive statistics like mean and standard deviation was used to analyse the emotional intelligence and conflict management among the participants. Regression analysis was used to find the correlation between the variables.

**Results:**

Most participants had a moderate level of emotional intelligence, with the mean and standard deviation (Mean—3.80, SD—0.67) and collaborating and compromise were the most often employed conflict resolution techniques with the mean 3.89 & SD 3.79, respectively. The least was employed of the competent style with mean 2.89. Emotional intelligence was found having significant positive relation with all the conflict management styles. Regarding the application of conflict management techniques, age group, years of experience, and educational background did not significantly differ from one another statistically with *p* = 0.05.

**Conclusion:**

The emotional intelligence of Saudi nurse managers affected all dispute resolution techniques. Programmes for nurse managers to develop their emotional intelligence may enhance their ability to resolve conflicts and function as an organisation.

## Introduction

The phrase “emotional intelligence” (EI) in management is not without controversy. But for professionals in the healthcare industry, especially nurse managers who must develop relationships to facilitate efficient management, having a solid understanding of emotional intelligence has become crucial (Liaqat et al., 2021). Emotional intelligence (EI) is a set of skills that people use to effectively control their emotions and stress. Self-awareness, self-regulation, motivation, empathy, and social awareness are its five constituent parts (Hussein, 2019). These elements support logical problem-solving techniques and long-lasting relationships. Additionally, they enhance patient outcomes in medical facilities (Ravanbakhsh Esmaeili et al., 2015).

Nurse managers with higher emotional intelligence are more able to maximize team commitment, productivity, and satisfaction to accomplish their goals (Salem et al., 2018). They also have an enhanced capacity to address challenges that emerge, such as miscommunication and workplace conflicts.

Conflicts are a serious issue in nursing environments. Myriad factors increase the likelihood of conflict, including organizational complexity, differing role expectations, decision-making process constraints, competition for scarce resources, undefined job boundaries, and personality differences (Labrague et al., 2018). Unresolved conflicts can prove expensive for healthcare facilities and may cause staff turnover (Başoğul and Özgür, 2016). Managing conflict is thus vital to ensure stability and aid nurse managers in finding appropriate solutions in healthcare facilities (Liaqat et al., 2021).

Reducing dysfunction and increasing productive ways to resolve disputes are key components of conflict management (Hussein, 2019.). According to Maniagigo and Alamri (2020), there are five widely accepted conflict management styles: cooperating, accommodating, competing, avoiding, and compromise (Maniago and Alamri, 2020). Therefore, rather of always depending on one method of conflict management, nurse managers should use approaches that are appropriate for the particular scenario and the stakeholders involved. Emotional intelligence (EI) seems to play a significant role in predicting the choice of conflict resolution techniques and situational reactions (Rafiq, 2022).

Quality is one aspect of quality evaluation for healthcare organizations (Ravanbakhsh Esmaeili et al., 2015), it is important to have the skills to regulate their emotions and deal with conflict effectively (Liaqat et al., 2021). Healthcare organizations that prioritize EI development may foster a more inclusive and peaceful work environment. In Saudi Arabia, Nurses play a vital role in the health care institutes. Nurses at various levels like Assistant head nurse, head nurse, incharge nurse, nursing director and nursing supervisors were involved in patient care. They engage in the care of patients at various circumstances and accordingly their management styles differs.

Studies and theories revealed that high EI enhanced prosocial discourse, conflict de-escalation, and solutions discourse—unit functioning and staff well-being skills (Başoğul and Özgür, 2016; Nikitara et al., 2024). From the cross-cultural point of view, EI also crossed over with predominant norms and beliefs; harmony-based, cooperative norms can lead to the application of integrative problem solving if leaders were high on EI (Gunkel et al., 2016; Llego et al., 2025).

Conflict on the nursing floors emanates from role ambiguity, workload, relations between professions, and rivalry over limited resources. Rahim’s model arrayed conflict-management styles along the dimensions of concern with oneself and concern with others—collaborating, compromising, accommodating, avoiding, and competing—so that collaboration remained the constructive, information-rich response to the very highly complex health issues (Rahim, 2023; Al-Hamdan et al., 2011). Empirical studies of nursing paired high EI with increased usage of integrative patterns (collaborating/compromising) and decreased usage of confrontive or withdrawing methods (Al-Hamdan et al., 2018). But cross-cultural research revealed that preferences were a function of the country culture and the work settings (Gunkel et al., 2016), which confirmed the value of setting-specific evidence.

In the Middle East, regional research reported correlations between EI and constructive conflict styles by nurse manager, estimates that were institution- and role-structure-specific (Al-Hamdan et al., 2018; Llego et al., 2025). More recent Saudi research highlighted EI as a leaders’ resource, reporting by managerial level and service line (Al-Hamdan et al., 2018; Llego et al., 2025). To date, despite these advances, little specificity remained on which of the many dimensions of EI (e.g., self-awareness, emotion managing, empathy, social skills, motivation) were highly correlated with the different conflict styles within cohorts of nurse managers by demographic background and role attributes, nor by which these correlations were diminished, enhanced.

There have been only limited attempts to delineate relationships between aspects of emotional intelligence and different conflict management styles, and the mechanisms that might drive this relationship. Clarifying these relationships may be beneficial for nurse managers who require evidence-based methods to handle complex conflict situations (Al-Hamdan et al., 2014). The present research aims to study how emotional intelligence (EI) impacts conflict management styles among Saudi nurse managers. It evaluates the EI-conflict management relationship and explores demographic influences (age, education, experience) on conflict management in Saudi governmental hospitals.

## Materials and methods

### Design and setting

The study also utilized a quantitative cross-sectional and correlational research design. The study was conducted between November 2023 to January 2024. Data were collected amongst diverse levels of Native Nurse Managers of Saudi Governmental hospitals spread across five regions of the country (Eastern, Western, Central, Southern, Northern). Heterogeneous sampling made efforts to have representative, accurate results with minimal bias.

### Sampling protocol

Saudi National Nurse Managers who were working with the Government Hospital, Saudi for more than 1 year were included in the study. Nurse Managers who were non-nationals and were working with the Government hospital were not included in the study.

A convenience sampling design was used to sample 500 nurse managers. Sampling calculated an appropriate sample size by taking into account the entire population of the Saudi nurse managers, which has an estimate of 13,000. The population has a 50% response distribution. With a 5% margin of error and a 98% confidence level, 500 nurse managers were calculated as appropriate to detect the expected effects. The formula for sample size calculation is presented below:


$$\text{Unlimited Population}:n=\frac{z^{2}\times \hat{p}(1-\hat{p})}{\varepsilon^{2}}$$

$$\text{Finite Population}:n\prime =\frac{n}{1+\frac{z^{2}\times \hat{p}(1-\hat{p})}{\varepsilon^{2}N}}$$

*n*, Sample size; *z*, Critical value = 1.960 with the selected confidence level of 98%; *N*, Population size = 13,000; *P*, Sample proportion (that describes the number of people in a sample who have a certain trait or characteristic), ranging from 50 to 70% to equal 0.63. Sample size was calculated using online sample size calculator. The calculated sample size for the study was 500.

### Data collection

Data was collected using the online questionnaire to find the relationship between EI and conflict management styles. The questionnaire consisted of three sections. The first requested demographic data with eight questions regarding participants’ gender, nationality, age, educational background, region, type of institution, current job title, and years of experience.

The second and third sections contained two tools that were adapted and translated to be suitable for the target population. Rahim Organizational Conflict Inventory-II (Rahim and Nace, 1994) (ROCI-II; Rahim, 1983) was used to measure the nature of conflicts. Prior permission was obtained to use the tool. The instrument is a standardized instrument in English that does not need translating by the nurse who takes part in the study. The scale has 28 items that measure how the respondent manage interpersonal conflict with subordinates. It has five sub-scales that include collaborating (7 items), competing (6 items), accommodating (5 items), avoiding (6 items), and compromising (4 items). Participants were asked to rate every statement on a 5-item Likert scale that had a score ranging between 1 (strong disagreement) and 5 (strong agreement). Scores indicated the level to which the respondents adhere to one of the conflict management styles that were identified.

The third component of the instrument was the Emotional Intelligence Scale (EIS; Vanderpol, 2001). Permission beforehand was given to utilize the instrument which doesn’t need translation. It consists of 25 items, five items per five dimensions of the scale, that is, self-awareness, managing emotions, motivating oneself, empathy, and social skills. The scale also consists of a 5-item Likert scale with ratings from 1 (very low ability) to 5 (very high ability). Possible scores range between 25 to 125, where a score of 101–125 indicates high EI, 50–100 indicates moderate EI, and the score lower than 50 indicates low EI.

Validity was assessed through the scales’ internal consistency by examining the correlation between the two scales’ total scores and their respective subsections, and reliability was assessed using Cronbach’s alpha. It was found that the tools are reliable at the alpha coefficient value 0.884 for conflicts scale and 0.960 for EI scale showing that both the scales were with high internal consistency and reliable.

The online questionnaire was distributed to the participants via email. The study link and invite comprised details of the study purpose, the right to confidentiality of the participants, voluntary participation by the members, and the estimated time to fill out the questionnaire. Participants were given the contact of the lead investigator in the event that they needed clarifications on the study. Once the target number of 500 responses were attained, the investigator closed the link of the questionnaire.

### Ethical considerations

Significant attention was given to ethical research standards throughout the study. Ethical approval for distributing the questionnaire was obtained from the Institutional Review Board (IRB) from all regions of Saudi Arabia, using the following reference numbers: Western region, IRB#(24-002), central region, IRB#(H-04-Q-001), Southern region, IRB#(REC-E1-1-2024), and Eastern region, IRB#(115-EP-2023).

## Data analyses

Descriptive statistics included frequencies, percentages, means, and standard deviations. Correlation and regression analyses validated measures and examined sub dimensions of emotional intelligence (EI) and conflict management styles. The data was analysed with the hypotheses that there exists a positive relation between the emotional intelligence and conflict management styles among nurses. The relation was analysed using the Karl Pearson Correlational Coefficient test. The second hypotheses framed was that there exists significant association between the demographic variables and the conflict management styles of the participants, which was assessed using ANOVA F test.

## Results

Data from 500 participants were analyzed using SPSS version 26 with a significance level of *p* = 0.05. Demographic variables, EI, and conflict management styles were analyzed via correlation and regression. With the total of 500 participants for inclusion Table 1 outlines the demographic characteristics of the sample: 28% male and 72% female participants. The majority (47%) are aged 31–40 years. 55% hold bachelor’s degrees in nursing. Regionally, 29% are from the Eastern, 25% from the Central, 16% from the Southern, 15% from the Northern, and 15% from the Western regions. Job titles include 33% assistant head nurses, 25% head nurses, 17% in-charge nurses, 16% nursing supervisors, and 9% nursing directors. Experience-wise, 46% have >5 years, 45% have 2–3 years, and 9% have 4–5 years.

**Table 1:** Description of the demographic variables of the participants (*N* = 500).

Variables	Categories	*N*	%
Gender	Male	140	28
	Female	360	72
Total		500	100
Nationality	Saudi	500	100
Total		500	100
Age	20–30	112	22
	31–40	234	47
	41–50	129	26
	51–60	24	5
	Over 60	1	0
Total		500	100
Educational background	Diploma in Nursing	109	21
	Graduate Certificate in Nursing	64	13
	Bachelor Degree in Nursing	276	55
	Master Degree in Nursing	48	10
	Doctoral Degree in Nursing	3	1
Total		500	100
Region	Central	126	25
	Eastern	142	29
	Northern	77	15
	Southern	79	16
	Western	76	15
Total		500	100
Hospital/institution type	Governmental	500	100
Total		500	100
Current job title	Assistant head nurse	164	33
	Head nurse	125	25
	Incharge nurse	87	17
	Nursing director	43	9
	Nursing supervisor	81	16
Total		500	100
Years experience	2–3 years	225	45
	4–5 years	46	9
	More than 5 years	229	46
Total		500	100

Table 2 presents mean values for emotional intelligence sub-dimensions (3.77 to 3.82, average 3.80, SD 0.67), indicating high overall EI ability in the sample.

**Table 2:** Description of the emotional intelligence of the participants under subdivisions of the scale (*N* = 500).

Sub-dimensions	Number and percentage	Very slight ability	Slight ability	Moderate ability	Much ability	Very much ability	Mean	Std. deviation
Self-awareness	*N*	5	19	97	230	149	3.77	0.73
	%	1	4	19	46	30		
Managing emotions	*N*	3	17	89	237	154	3.81	0.72
	%	1	3	18	47	31		
Motivating yourself	*N*	4	14	94	245	143	3.80	0.71
	%	1	3	19	49	29		
Empathy	*N*	4	18	91	245	142	3.78	0.72
	%	1	4	18	49	28		
Social skills	*N*	5	17	89	228	161	3.82	0.75
	%	1	3	18	46	32		
Total	*N*	4	18	93	249	136	3.80	0.67
	%	1	4	19	50	27		

Table 3 shows 32.2% of Saudi nurse managers have high EI (mean 112.9, SD 7.6), 66.8% moderate (mean 87.1, SD 10.9), and 1% low (mean 33, SD 10.6).

**Table 3:** Description of the level of emotional intelligence among the participants (*N* = 500).

	Level of EI	*N*	%	Mean	Std. deviation	Min
Emotional intelligence	Low EI	5	1	33	10.6	25
	Moderate EI	334	66.8	87.1	10.9	50
	High EI	161	32.2	112.9	7.6	101

Table 4 outlines conflict management styles: the mean values of each styles are as follows: Collaborating (3.89) was most common, followed by compromising (3.79) and accommodating (3.41). Avoiding (3.17) and competing (2.89) styles were least frequent. Inorder to test the hypothesis that the Saudi nurse managers’ EI significantly impact the used conflict management styles, regression analysis was used with the formula 𝑦= 𝑏0+ 𝑏1𝑋1 + ℇ. Considering 𝑦, dependent variable (conflict management style); 𝑏0, constant; 𝑋1, independent variable (emotional intelligence); 𝑏1, the coefficient for (emotional intelligence).

**Table 4:** Description of the conflict management styles of the participants (*N* = 500).

Sub-dimensions	Number and percentage	Fully disagree	Disagree	Moderately	Agree	Fully agree	Mean	Std. deviation
Collaborating	*N*	10	22	90	193	185	3.89	0.81
	%	2	4	18	39	37		
Accommodating	*N*	5	41	194	218	42	3.40	0.64
	%	1	8	39	44	8		
Competing	*N*	50	135	130	144	41	2.89	0.92
	%	10	27	26	29	8		
Avoiding	*N*	37	64	196	166	37	3.17	0.83
	%	7	13	39	33	7		
Compromising	*N*	16	18	111	192	163	3.79	0.81
	%	3	4	22	38	33		
Conflict management style	*N*	7	23	193	246	31	3.43	0.55
	%	1	5	39	49	6		

Table 5 describes that emotional intelligence had an influence on all the conflict management styles, as *r* = 0.589 (*p* < 0.01). Also, emotional intelligence was positively and significantly correlated with collaborating style (*r* = 0.739, *p* < 0.01), accommodating style (*r* = 0.556, *p* < 0.01), avoiding style (*r* = 0.233, *p* < 0.05), and compensating style (*r* = 0.611, *p* < 0.05), while emotional intelligence was not significantly correlated with competing style (*r* = −0.022, *p* > 0.01). The first hypothesis saying that there exists a significant relation between the emotional intelligence and conflict management styles of nurses was tested using Karl Pearson’s Correlation and was found accepted.

**Table 5:** Description of correlation of emotional intelligence on conflict management styles.

Sub-dimensions	Correlation coefficient	Collaborating	Accommodating	Competing	Avoiding	Compromising	Conflict management style
Self-awareness	Pearson correlation	0.685	0.512	−0.016	0.210	0.550	0.540
Managing emotions	Pearson correlation	0.656	0.469	−0.025	0.199	0.564	0.520
Motivating yourself	Pearson correlation	0.688	0.514	0.024	0.224	0.575	0.567
Empathy	Pearson correlation	0.659	0.549	−0.010	0.242	0.557	0.555
Social skills	Pearson correlation	0.737	0.534	−0.071	0.207	0.591	0.554
Emotional intelligence	Pearson correlation	0.739	0.556	−0.022	0.233	0.611	0.589

Table 6 clarifies the correlation between demographic constructs and conflict management Conflict management Conflict management styles were not significantly observed to be correlated with years of experience, *F* = 1.126, *p* = 0.325. Also, no correlation of conflict management styles with education level, *F* = 1.293, *p* = 0.272.

**Table 6:** Description of the impact of demographic variables with the conflict management styles.

Variable	Categories	*N*	Mean	Std. deviation	*F*	*P*
Age	20–30	112	3.444	0.576	1.323	0.260
	31–40	234	3.386	0.530		
	41–50	129	3.520	0.555		
	51–60	24	3.487	0.560		
	Over 60	1	3.214	–		
Years of experience	2–3 Years	225	3.454	0.455	1.126	0.325
	4–5 Years	46	3.323	0.645		
	More than 5 Years	229	3.446	0.610		
Educational background	Diploma in Nursing	109	3.372	0.627	1.293	0.272
	Graduate Certificate in Nursing	64	3.345	0.655		
	Bachelor Degree in Nursing	276	3.450	0.495		
	Master degree in Nursing	48	3.523	0.516		
	Doctoral Degree in Nursing	3	3.694	0.482		

## Discussion

The present study was conducted to critically study the impact of EI on conflict management styles and how EI might assist nurse managers in Saudi Arabia. In this study, analysis of study participants’ questionnaire responses revealed that nurse managers had an overall moderate level of emotional intelligence. This finding may be explained by the fact that Saudi nurse managers need to develop their emotional intelligence abilities to get high emotional intelligence scores prior to becoming nurse managers, such that they are already well aware of their own and others’ emotions while working as nurse managers. Emotionally intelligent nurse managers are better equipped to control their emotions and find constructive solutions to problems. The findings contradict those of Salem et al. (2018), who indicated high emotional intelligence among nurse managers (Salem et al., 2018), but they are consistent with previous research by Al-Hamdan et al. (2011), Hussein (2019), and Al-Hamdan et al. (2014).

Emotional intelligence is the most significant indicator of success for a nurse manager. In this study, nurse managers tended towards high scores on all of the subdimensions of emotional intelligence. This finding is consistent with those of Baudry et al. (2018), who found high scores on all dimensions of emotional intelligence among 513 nurse managers. The EI subdimensions with the highest mean scores in this study were social skills, managing emotions, and motivating oneself. Study results indicate that Saudi nurse managers are capable of managing their emotions and can motivate themselves and others at work (Baudry et al., 2018). Moreover, nurse managers are well-prepared to manage any conflict during daily practice. While self-awareness and empathy showed the lowest scores among the EI subdimensions in this sample, they were still relatively high. Generally, Saudi nurse managers have abilities that enable them to respond to conflicts in an efficient manner (Al-Hamdan et al., 2014).

Conflict management styles have a direct impact on emotional intelligence, which can have numerous professional consequences (Gunkel et al., 2016). The results of this study revealed that the most frequently used conflict management styles among Saudi nurse managers were the collaborating and compromising styles. Data indicated that nurse managers use these strategies as part of a productive and positive work culture, solidifying values and attitudes that promote productive working relationships. Managers promote trust, respect, and the idea of mutual benefit. This, in turn, prompts managers to consider the needs and goals of other parties when conflicts arise. The study’s findings match those of Al-Hamdan et al. (2018), Chan et al. (2014), Salem et al. (2018), Al-Hamdan et al. (2014), and Mohamed and Yousef (2014). The styles used least frequently by Saudi nurse managers were the competing and avoiding styles, congruent with the results of Al-Hamdan (2009), Mohamed and Yousef (2014), and Al-Hamdan et al. (2014). Notably, this outcome contrasts with a finding commonly reported in studies with Western samples that the avoiding style is most frequently used for conflict management, perhaps indicative of a cultural difference (Başoğul and Özgür, 2016).

Regarding the relationship between nurse managers’ emotional intelligence and conflict management styles, results showed that EI was associated with all conflict management styles. There were positive, significant relationships found between emotional intelligence and collaborating, compromising, accommodating, and avoiding styles, while a negative association was found between EI and the competing style. The current findings match those of Al-Hamdan et al. (2014).

It was predicted that demographic characteristics would have an impact on conflict management styles. The results showed, however, that there were no statistically significant differences among nurse managers’ conflict management styles when comparing by age group, educational background, or years of experience. These results match findings by Al-Hamdan et al. (2014) though not those of Delak et al., who reported statistically significant differences in nurse managers’ conflict management styles according to gender, years of experience, and educational background (Al-Hamdan et al., 2014; Delak and Širok, 2022). Nevertheless, the present findings are clear that the conflict management styles most used by Saudi nurse managers, at all levels of experience and educational background, are the collaborating and compromising styles. In the 51–60 age range, Saudi nurse managers most frequently adopted a competitive attitude. The small sample size (*n* = 24) in this age range, however, might make it more difficult to interpret this result. To investigate how nurse managers select specific conflict resolution philosophies, more research is required using larger, representative sample sizes across a wide age range.

The current work focused on nurse leaders in the Saudi setting and conflict-management styles against the backdrop of that environment versus EI. Earlier syntheses have revealed international and cross-cultural regularities (Gunkel et al., 2016; Nikitara et al., 2024) and cross-cultural studies of diverse populations outside the Gulf region (Başoğul and Özgür, 2016). Nonetheless, works originating with the specific region have been very few, such as those by the Kingdom of Jordan (Al-Hamdan et al., 2018) and more recent assessment of the Kingdom of Saudi Arabia of the emotional intelligence and of the leadership (Llego et al., 2025). Our study filled the regional specific environment literature and validated Rahim’s conflict model applicable to the Kingdom of Saudi Arabia hospitals (Rahim, 2023).

Gains were especially large on social skills, emotion regulation, and self-motivation, with modest empathy and self-awareness. Compromising and coordinating were employed by far the most; competing and avoiding were relatively often used. This profile fell into the observed mode of nursing cohorts where high EI goes with integrative styles (Başoğul and Özgür, 2016) and Rahim’s cooperatively oriented, problem-based position (Rahim, 2023). The relatively modest overall EI of our sample disagreed with some Middle-Eastern descriptions of high EI (Al-Hamdan et al., 2018). Differences between institutions, between assessment instruments, or between sampling periods might account for the discrepancy, which aligns with diversity observed by the nursing review (Nikitara et al., 2024). Low usage of avoidance also differed from several Western samples collated by Nikitara et al. (2024), which might reflect a culture-specific preference by Saudi services for apparent, consensus-based forms of management. The theoretic contribution of the study was to locate the linkage between conflict and EI: on these occasions, EI was associated most highly with collaborative and compromising responses, confirming cross-cultural studies that EI predicts cooperative style of handling conflict (Gunkel et al., 2016) and nurse studies that associate EI with conflict competence (Başoğul and Özgür, 2016). In practice, pipelines to leaders can be augmented by a measure of EI and targeted development of it—specifically of the component of self-awareness and empathy—plus scenario practice at collaborative problem solving. These interventions follow the recommendation of the nurse synthesis (Nikitara et al., 2024) and of preliminary Saudi studies of EI and leaders’ behavior (Llego et al., 2025).

The collaborating style had the strongest correlation with emotional intelligence in the present study. This finding implies that Saudi nurse managers with higher emotional intelligence are more adept at resolving conflicts, and are more able to work together with others to address problems. Another implication is that nurse managers with higher emotional intelligence are more adept at handling problems in complex or strategic situations.

## Conclusion

Results show that among nurse managers, conflict management techniques are highly influenced by emotional intelligence. Four styles (collaborating, compromising, accommodating, and avoiding) showed positive relationships with emotional intelligence, whereas competing showed negative correlations. The majority of individuals showed modest emotional intelligence, indicating room for improvement with more instruction. The strategies of collaborating and compromising were preferred above competing. The subdimensions of emotional intelligence showed strong positive connections, especially in the areas of motivation, social skills, and emotion management. There was no discernible relationship between conflict management styles and demographic characteristics. As a result, the study suggests fostering an organisational culture, which facilitates nurse managers’ ability to resolve conflicts. As a result, the organisational culture and the nurses’ calibre of work performance are strengthened.

### Limitations

The study’s use of convenience sampling increases the risk of selection bias and reduces the results’ potential to be applied broadly. Results could therefore be skewed in favour of those who are more willing or easily accessible to participate. The cross-sectional methodology of the study, which only gathers data at one specific moment in time, makes it more challenging to establish causality or determine the direction of connections between the conflict management strategies used by Saudi Arabian nurse managers and their emotional intelligence. Longitudinal research designs would eventually offer a more complete understanding of these constructs.

## Data Availability

The original contributions presented in the study are included in the article/supplementary material, further inquiries can be directed to the corresponding author.
